# The Mycotoxin Deoxynivalenol Predisposes for the Development of *Clostridium perfringens*-Induced Necrotic Enteritis in Broiler Chickens

**DOI:** 10.1371/journal.pone.0108775

**Published:** 2014-09-30

**Authors:** Gunther Antonissen, Filip Van Immerseel, Frank Pasmans, Richard Ducatelle, Freddy Haesebrouck, Leen Timbermont, Marc Verlinden, Geert Paul Jules Janssens, Venessa Eeckhaut, Mia Eeckhout, Sarah De Saeger, Sabine Hessenberger, An Martel, Siska Croubels

**Affiliations:** 1 Department of Pathology, Bacteriology and Avian Diseases, Faculty of Veterinary Medicine, Ghent University, Merelbeke, Belgium; 2 Department of Pharmacology, Toxicology and Biochemistry, Faculty of Veterinary Medicine, Ghent University, Merelbeke, Belgium; 3 Department of Nutrition, Genetics and Ethology, Faculty of Veterinary Medicine, Ghent University, Merelbeke, Belgium; 4 Department of Applied Biosciences, Faculty of Bioscience Engineering, Ghent University, Ghent, Belgium; 5 Department of Bio-analysis, Faculty of Pharmaceutical Sciences, Ghent University, Ghent, Belgium; 6 Biomin Research Center, Tulln, Austria; The Ohio State University, United States of America

## Abstract

Both mycotoxin contamination of feed and *Clostridium perfringens*-induced necrotic enteritis have an increasing global economic impact on poultry production. Especially the *Fusarium* mycotoxin deoxynivalenol (DON) is a common feed contaminant. This study aimed at examining the predisposing effect of DON on the development of necrotic enteritis in broiler chickens. An experimental *Clostridium perfringens* infection study revealed that DON, at a contamination level of 3,000 to 4,000 µg/kg feed, increased the percentage of birds with subclinical necrotic enteritis from 20±2.6% to 47±3.0% (*P*<0.001). DON significantly reduced the transepithelial electrical resistance in duodenal segments (*P*<0.001) and decreased duodenal villus height (*P* = 0.014) indicating intestinal barrier disruption and intestinal epithelial damage, respectively. This may lead to an increased permeability of the intestinal epithelium and decreased absorption of dietary proteins. Protein analysis of duodenal content indeed showed that DON contamination resulted in a significant increase in total protein concentration (*P* = 0.023). Furthermore, DON had no effect on *in vitro* growth, alpha toxin production and *netB* toxin transcription of *Clostridium perfringens*. In conclusion, feed contamination with DON at concentrations below the European maximum guidance level of 5,000 µg/kg feed, is a predisposing factor for the development of necrotic enteritis in broilers. These results are associated with a negative effect of DON on the intestinal barrier function and increased intestinal protein availability, which may stimulate growth and toxin production of *Clostridium perfringens*.

## Introduction

Worldwide, necrotic enteritis (NE) leads to important production losses, increased feed consumption and mortality rates, and a reduced welfare of broiler chickens [1]–[4]. The causative agent of NE is *Clostridium perfringens*, a Gram-positive spore forming bacterium which occurs ubiquitously in the environment, in feed and in the gastrointestinal tract of animals and humans [5], [6]. It has been suggested that alpha toxin production is an essential virulence factor in the pathogenesis of NE [7], but recently it was established that only strains producing NetB toxin, a β-pore-forming toxin, are capable of inducing NE in broiler chickens under specific conditions that predispose to the disease [8], [9].

Acute NE is characterized by a sudden increase in mortality, often without premonitory symptoms. Nowadays, the subclinical form is becoming more prevalent, and is mainly characterized by intestinal mucosal damage without clinical signs or mortality. This leads to a decreased digestion and absorption of nutrients, a reduced weight gain and an impaired feed conversion rate [4], [8].

Notwithstanding the role of *C. perfringens* in poultry production losses, the mere presence of virulent strains in the intestinal tract of broilers, or even the inoculation of chickens with high doses of these strains, does not always lead to the development of NE. Predisposing factors including dietary, husbandry and immune factors [7], [10], [11], are required to reproduce the disease [12]–[14]. The best-known predisposing factor is mucosal damage caused by coccidial pathogens [13], [15], which could provide *C. perfringens* with essential nutrients and thus stimulate massive overgrowth [16], [17]. *C. perfringens* is lacking many genes of the orthologous enzymes required for amino acid biosynthesis, among others for arginine, phenylalanine, tryptophan, tyrosine, histidine, leucine, isoleucine, valine, glutamate, lysine, methionine, serine and threonine. Therefore, *C. perfringens* growth is restricted in an environment where the amino acid supply is limited [16], [18], [19].

The mycotoxin deoxynivalenol (DON) is one of the most common contaminants in poultry feed worldwide. DON is a type B trichothecene produced by among others *Fusarium (F.) graminearum* and *F. culmorum*. Recent data on global mycotoxin occurrence showed that 59% of 5,819 samples of animal feed tested positive for the presence of DON. The average contamination level was 1,104 µg DON/kg feed, with a maximum observed level of 49,307 µg/kg [20]. The European maximum guidance level for poultry feed is set at 5,000 µg/kg feed [21].

Poultry is considered rather tolerant to DON. It has been suggested that concentrations higher than 5,000 µg/kg feed are necessary to negatively influence the growth performance of broilers [22], [23]. This mycotoxin acts as an inhibitor of the protein synthesis at the ribosomal level whereby rapidly proliferating cells in tissues with high protein turnover rates, such as the immune system and small intestine, are most affected [23]. Accordingly, DON negatively influences small intestinal epithelial cell integrity and morphology [24]–[29]. As a consequence of the negative effect of DON on the gastro-intestinal epithelial cells, feeding DON-contaminated diets can lead to greater susceptibility to enteric infections [27]. Only few studies have investigated the interaction between DON and enteric pathogens. In pigs, it has been shown in an intestinal ileal loop model that co-exposure to DON and *Salmonella* Typhimurium potentiates the inflammatory response in the gut [30]. *In vitro*, intestinal porcine epithelial cells (IPEC-1) show an increased translocation of a septicemic *Escherichia coli* (O75:K95) after DON exposure [26]. It is hitherto unclear whether the intestinal epithelial damage caused by contamination levels of DON below 5,000 µg/kg in feed, may act as an additional predisposing factor in broiler NE. We hypothesized that this intestinal damage may lead to higher protein availability for clostridial proliferation in the small intestine.

The objectives of this study were to examine whether DON at concentrations in the feed below the EU maximum guidance level predisposes for NE in broilers, and to gain insights in the mechanisms responsible for this interaction. Therefore, the effects of DON on the intestinal epithelial barrier function and on intestinal protein availability for clostridial proliferation were evaluated. Also, the direct effect of DON on *in vitro* bacterial proliferation, alpha toxin production and *netB* transcription was studied.

## Materials and Methods

### Deoxynivalenol

For the *in vitro* assessment of the impact of DON on growth and toxin production of *C. perfringens*, a DON stock solution of 2000 µg/mL (Fermentek, Jerusalem, Israel) was prepared in anhydrous methanol and stored at −20°C. Next, serial dilutions of DON were prepared in tryptone glucose yeast (TGY) broth medium.

For the animal trials, DON was produced *in vitro* from cultures of *F. graminearum* in accordance to the protocol described by Altpeter *et al.* [31] (Romer Labs, Tulln, Austria), and was mixed into the experimental feed.

### Bacterial strains

*C. perfringens* strain 56 has been used previously to induce NE in an *in vivo* model in broilers [6], [32]. Originally this strain was isolated from the gut of a broiler chicken with severe NE lesions, and characterized as a *netB* toxin positive type A strain (no β_2_ or enterotoxin genes) as well as a producer of moderate amounts of alpha toxin *in vitro* [33].

In addition to strain 56, a *netB* toxin negative strain (*C. perfringens* strain 6 [33]) was included as negative control for *in vitro netB* transcription measurement.

#### Birds and housing

Non-vaccinated Ross 308 broilers were used that were obtained as one-day-old chicks from a commercial hatchery. Each group consisted of approximately equal numbers of males and females. All treatment groups were housed in the same room, in cages of 1.44 m^2^, on a litter floor. All cages were separated by solid walls to prevent direct contact between birds from different treatment groups. Before each trial, the cages were decontaminated with peracetic acid and hydrogen peroxide (Hygiasept vaporizer climasept; SARL Hygiasept, Sevrey, France) and a commercial anticoccidial disinfectant (Bi-OO-Cyst Coccidial Disinfectant; Biolink, York, United Kingdom).

Chickens had *ad libitum* access to drinking water and feed and were subjected to a 23 h/1 h light/darkness programme. The animals were not fasted before euthanasia. The environmental temperature was adjusted to the changing needs of the animals according to their age (week 1∶35°C, week 2∶30°C, week 3∶25°C).

#### Feed

All birds were given a starter diet during the first eight days of the experiment, and subsequently a grower diet until the end of the trial. The diet was wheat:rye (43%:7.5%) based, with soybean meal as the main protein source during the first 16 days. From day 17 onwards, the same grower diet was used with the exception that fishmeal (30%) was added as protein source instead of soybean meal. Further details of the feed composition were as previously described [32].

In the exposed groups, an artificially DON contaminated diet was fed from day 1 onward. The contaminated feed was produced by adding DON to a control diet. To test for DON concentrations in the feed, samples were taken at three different locations in the batch and subsequently pooled. All diets were analysed for the content of DON and other mycotoxins with a validated multi-mycotoxin liquid chromatography-tandem mass spectrometry method (LC-MS/MS) [34]. The levels of DON in the different batches of control feed was below the limit of quantification. The DON contamination level in the different batches of contaminated feed varied between 2,884±800 µg/kg and 4,384±1,300 µg/kg feed. All other mycotoxins tested were either absent or present in low concentrations. Table S1 shows the different mycotoxins tested, their limit of detection (LOD) and limit of quantification (LOQ) and their concentration in the different feeds.

#### Animal experiment 1: *C. perfringens* experimental infection study

The trial was performed following an adapted protocol based on a previously described experimental infection model, with the modification that no coccidial challenge was administered [32]. In the trial, 360 chicks were divided into 4 experimental groups, each group consisting of 3 cages of 30 chicks. The experimental groups are described in Table 1 One group was experimentally infected with *C. perfringens* and received a control diet. A second group was experimentally infected with *C. perfringens* and received a DON contaminated diet, while a third group was fed a DON contaminated feed but did not receive *C. perfringens*. A fourth group was a negative control (no *C. perfringens* and control feed). Gumboro vaccine (Nobilis Gumboro D78, MSD Animal Health, Brussels, Belgium) was administered in the drinking water on day 16 to all birds. Experimental infection with *C. perfringens* consisted of oral inoculation of the birds with 4.10^8^ cfu of *C. perfringens* strain 56 at days 17, 18, 19 and 20. The bacteria for the animal experiment were cultured anaerobically overnight in brain heart infusion broth (BHI, Oxoid, Basingstoke, UK) supplemented with 0.375% glucose at 37°C. The actual number of bacteria/mL was assessed by plating tenfold dilutions on Columbia agar (Oxoid) with 5% sheep blood, incubated anaerobically overnight at 37°C. Birds that were not infected with *C. perfringens* received a sham inoculation with BHI broth.

**Table 1:** Experimental groups and impact of DON on the number of chickens affected by necrotic enteritis (NE) and bodyweight (BW) gain.

					mean daily BW gain (g/day)(2)		
GROUP	DON	*C. perfringens*	percentage of animals with NE lesions (%)		day 1–7	day 8–14	day 15– euthanasia
**Cp** (1) **alone**	−	+	20±2.6^a^	♂	20±7	38±7	53±23
				♀	19±2	37±4	44±21
**Cp+DON** (1)	+	+	47±3.0^b^	♂	18±3	41±8	54±24
				♀	20±6	44±5	56±19
**DON** (1) **alone**	+	−	0±0.0(3)	♂	18±4	39±7	60±14
				♀	17±5	35±6	44±10
**negative control**	−	−	0±0.0(3)	♂	19±2	44±6	65±19
				♀	17±5	38±6	67±17

Four experimental groups were included, of which each experimental group consisted of 3 cages of 30 chickens. After a feeding period of 3 weeks chickens were euthanized.

(1) Cp: *C. perfringens* challenge strain 56; DON: deoxynivalenol challenge.

(2) Results bodyweight (BW) gain based on ten animals per group in triplicate.

(3) Since the mean NE lesion score in groups DON alone and negative control was zero, both groups were excluded from statistical analysis with respect to macroscopic NE lesion scoring.

a–b significantly different within one column (*P*<0.05). All data are presented as mean ± standard deviation.

On days 21, 22 and 23, each day one third of each group was euthanized and the birds were immediately submitted to necropsy. Single-blind macroscopic NE lesion scoring of the small intestine (duodenum to ileum) was performed as previously described by Keyburn *et al.* [7]. Birds with lesion scores of 2 or more were classified as NE positive.

In addition, contents of three small intestinal segments of 27 birds per group of the third (DON, no *C. perfringens*) and fourth (negative control) experimental group were collected and stored at −20°C until further use for protein analysis. The three segments were duodenum, jejunum and ileum. The duodenum was defined as the segment encompassing the duodenal loop, whereas the jejunum was defined as the segment between the end of the duodenal loop and Meckels diverticulum. The ileum comprised the distal segment starting at Meckels diverticulum and ending at the ileo-cecal junction.

Contents of these three segments were used to determine intestinal nitrogen (N) concentration by the Kjeldahl method used for feeding stuffs (ISO2005). Percentage crude protein per dry matter of the intestinal content was calculated from Kjeldahl N values, using 6.25 as conversion factor to protein level.

The bodyweight (BW) of 30 identified chickens per experimental group was measured at day 1, 7, 14 and at the day of euthanasia. Bodyweight gain was determined as the differences in BW divided by the period of time. The presence of coccidiosis was excluded by faecal oocyst count and macroscopic coccidiosis lesion scoring of the intestines [35].

#### Animal experiment 2: Effect of DON on villus height and transepithelial electrical resistance

Eighteen birds were divided into 2 experimental groups, each group consisting of 3 cages of 3 chicks. One group was fed a control diet, and the other group was fed a DON-contaminated diet. All birds received Gumboro vaccine on day 16 and they all received a sham inoculation with blank BHI broth on day 17, 18, 19 and 20. On day 21, immediately after euthanasia of the animals, 1 cm samples from the mid-duodenum, mid-jejunum and mid-ileum were collected for evaluation of the intestinal morphology. These samples were fixed in neutral-buffered formalin, and processed afterwards using standard protocols for hematoxylin and eosin staining of paraffin sections. Villus height and crypt depth were measured using a light microscope with Leica LAS software (Leica Microsystems, Diegem, Belgium). The average of 5 to 15 measurements per segment per animal was calculated.

The remainder of the mid-duodenal segment was immersed into oxygenated (O_2_/CO_2_, 95/5%) Krebs Henseleit buffer solution (Sigma-Aldrich) of pH 7.4. Before opening the intestinal segment, the underlying serosal layer was stripped off. Segments were opened along the mesenteric border and rinsed with buffer solution. Per chicken, three duodenal segments of 2 cm in length were cut and each mounted in an Ussing chamber (Mussler Scientific Instruments, Aachen, Germany). Epithelial sheets had an exposed surface area of 0.28 cm^2^. Mucosal and serosal compartments were simultaneously filled with 7 mL Krebs Henseleit buffer. Four Ag/AgCl electrodes were connected to each chamber by 3M KCl-agar bridges. The electrodes were coupled to an external six-channel microcomputer controlled voltage/current clamp. After an equilibration period of 30 minutes, the transepithelial potential difference (PD, mV) and transepithelial electrical resistance (Rt - TEER, Ω.cm^2^) were monitored as measures of tissue viability and integrity, respectively, with the tissue unclamped in open circuit mode. Current (Isc, µA/cm^2^) was calculated from Ohm’s law using the following equation Isc = Pd/Rt [36].

The *in vivo* experimental protocols and care of the animals were approved by the Ethical Committee of the Faculty of Veterinary Medicine, Ghent University, Belgium (EC 2011/169 EC 2012/074).

### *In vitro* study of the effect of DON on *C. perfringens* growth, alpha toxin production and *netB* transcription

Following concentrations of DON were tested for its effect on *C. perfringens* growth, alpha toxin production and *netB* transcription: 0, 0.2, 2 or 20 µg DON/mL TGY medium.

*C. perfringens* strains 6 and 56 were grown for 24 h in TGY broth medium. Subsequently, this bacterial culture was 1∶1000 diluted in the different DON concentrations and incubated anaerobically at 37°C. Clostridial growth curve was assessed by bacterial plating of a ten-fold dilution series at 0, 2, 3, 4, 5, 6, 7, 8 and 24 h after inoculation. Ten-fold dilutions were made in phosphate buffered saline (PBS) solution. Six droplets of 20 µL of each dilution were plated on Columbia agar with 5% sheep blood. After anaerobic incubation overnight at 37°C, the number of colony forming units (cfu)/mL was determined by counting the number of bacterial colonies for the appropriate dilution.

Quantitative detection of alpha toxin in the *C. perfringens* (strain 56) culture supernatants was performed as previously described by Gholamiandekhordi *et al.*, using the Bio-X Alpha Toxin Elisa Kit (Bio-X Diagnostics, Jemelle, Belgium) [33]. Positive (pure alpha toxin) and negative controls (incubation buffer) were included. All tests were performed in triplicate with two technical repeats in each experiment. Subsequently, the mean optical density (OD) value was calculated relative to the positive control value, which was set at 1.

The impact of DON on *netB* transcription was tested by qRT-PCR [37]. The transcription levels of *netB* in the presence of DON were compared to non-DON contaminated test conditions normalized to the housekeeping gene *rpoA*, encoding RNA polymerase subunit A. One mL of mid and late logarithmic growth phase was collected for all test conditions, as described above, from three biological replicates. Based on the growth curve, mid and late logarithmic growth phase were defined after 3 h and 6 h incubation, respectively. Cells were collected by centrifugation at 9,300×g for 5 min at 4°C. Total RNA was isolated using RNAzol RT (Sigma-Aldrich, Bornem, Belgium) and 40 ng of RNA was converted to cDNA with iScript cDNA Synthesis Kit (Bio-rad, Nazareth Eke, Belgium) in accordance with the manufacturer’s instructions. RT-qPCR was performed using SYBR-green 2x master mix (Bioline, Brussels, Belgium) in a Bio-Rad CFX-384 system. Each reaction was done in triplicate in a 12 µL total reaction mixture using 2 µL of the cDNA sample and 0.5 µM final qPCR primer concentration (Table S2). The q-PCR conditions used were 1 cycle of 95°C for 10 min, followed by 40 cycles of 95°C for 30 s, 60°C for 30 s, and stepwise increase of the temperature from 65° to 95°C (at 10s/0.5°C). Melting curve data were analysed to confirm the specificity of the reaction. For construction of the standard curve, the PCR product was generated using the standard PCR primers listed in Table S2 and DNA from *C. perfringens*. After purification (MSB Spin PCRapace, Stratec Molecular, Berlin, Germany) and determination of the DNA concentration with a Nanodrop ND 1000 spectrophotometer (Nanodrop Technologies, Wilmingtom, DE, USA), the concentration of the linear dsDNA standard was adjusted to 1×10^8^ to 1×10^1^ copies per µL with each step differing by tenfold. The copy numbers of samples were determined by reading off the standard series with the Ct values of the samples.

### Statistical analyses

Statistical program SPSS version 21 was used for data analysis. All *in vitro* and *in vivo* experiments were conducted in triplicate with three repeats per experiment, unless otherwise noted. To compare the number of NE positive birds (lesion score ≥2) between different groups, binomial logistic regression was used. Bodyweight gain was analysed by using an univariate general linear model. Total protein levels, electrophysiological parameters, villus height and crypt depth measurements, *in vitro* assessment of clostridial growth and toxin production, were assessed by independent t-test, after determination of normality and variance of homogeneity. Significance level was set at 0.05.

## Results

### Animal experiments

#### DON significantly increases the number of chickens affected by NE

**Figure 1:**
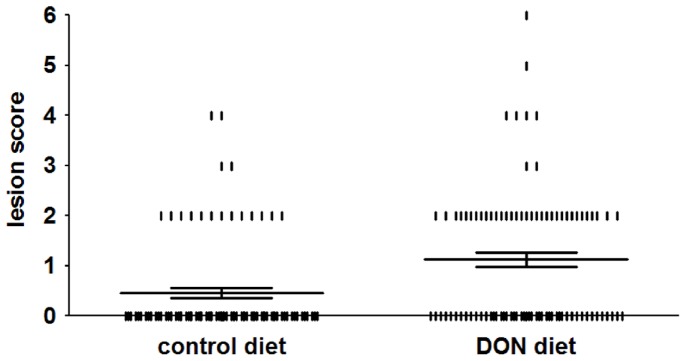
Lesion scores of individual broiler chickens challenged with *C. perfringens*. Chickens were fed either a control or DON-contaminated diet and subsequently challenged with *C. perfringens* strain 56. The solid bars represent the average lesion score in each group. Error bars represent SEM. Intestinal lesions in the small intestine (duodenum to ileum) were scored as previously described [7]; 0 no gross lesions; 2 small focal necrosis or ulceration (one to five foci); 3 focal necrosis or ulceration (six to 15 foci); 4 focal necrosis or ulceration (16 or more foci); 5 patches of necrosis 2 to 3 cm long; 6 diffuse necrosis typical field cases. The score 1 used for congested intestinal mucosa was not applied here because of difficulties in scoring this characteristic objectively, and due to the lack of scientific documentation of an association between “congested intestinal mucosa” and necrotic enteritis. Birds with lesion scores of 2 or more were classified as NE positive.

The DON-contaminated diet led to a significantly increased number of chickens with NE; i.e. 20±2.6% of the chickens in the group inoculated with *C. perfringens* and fed a control diet were positive for NE lesions, while in the group inoculated with *C. perfringens* and fed a DON-contaminated diet 47±3.0% of the broilers were positive (*P*<0.001) (Figure 1, Table 1). No animals with NE lesions were detected in the groups without bacterial challenge. Lesion scores of individual broiler chickens challenged with *C. perfringens* are shown in Figure 1. In NE positive chickens the lesions were mainly observed in the duodenum (29±0.1% and 31±0.1% of the NE positive chickens in the control and DON group, respectively) and jejunum (94±0.1% and 96±0.1%, respectively). In the ileum, only one animal in the control group and no animals in the DON group showed lesions (score 2). No statistically significant differences were observed in BW gain between the different groups (Table 1). No coccidia challenge was observed, since *Eimeria* oocysts were absent in the excreta and no macroscopic coccidiosis lesions were observed.

#### DON increases the intestinal protein concentration

**Figure 2:**
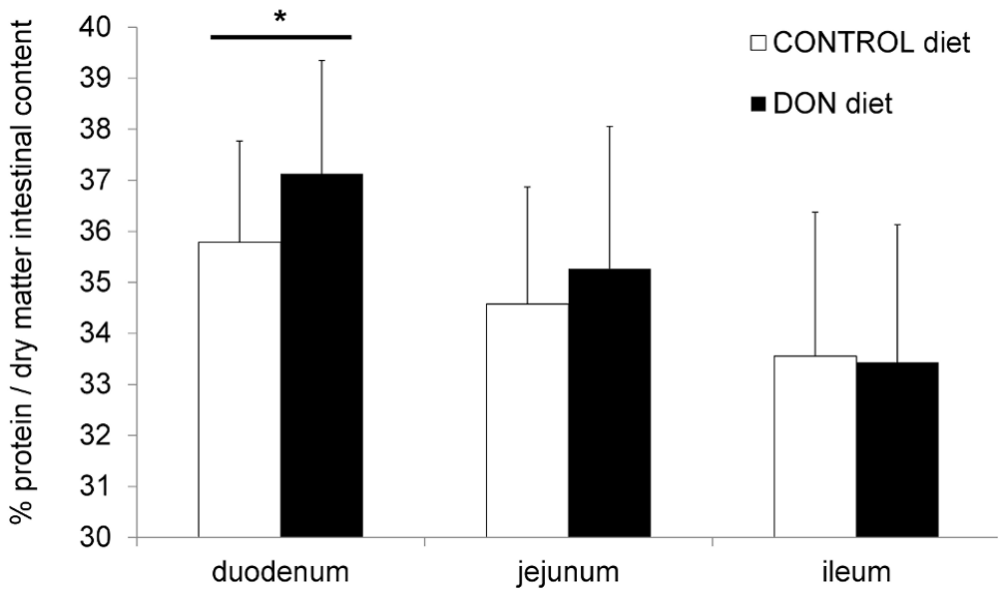
Protein concentration in intestinal content is significantly increased in duodenum of chickens fed a DON-contaminated diet. Percentage crude protein per dry matter of the intestinal content was determined by the Kjeldahl method. Results are presented as the mean protein level of 27 samples per group per intestinal segment. Error bars represent SD. ^(*)^ significantly different (*P*<0.05) within one intestinal segment.

The total protein concentration in duodenal intestinal content was significantly higher in chickens fed the DON contaminated diet (*P* = 0.023). However, no effect of DON on the total protein concentration in jejunal and ileal intestinal content was detected (Figure 2).

#### DON reduces transepithelial electrical resistance in duodenal segments

No difference was observed between the control and the DON group for PD (−2.7±0.14 and −2.3±0.13 mV, respectively) and Isc (7.0±0.38 and 6.4±0.36 µA/cm^2^, respectively), but TEER was significantly lower (*P*<0.001) for DON fed birds (369.8±5.47 Ω.cm^2^) compared with the control birds (392.2±4.72 Ω.cm^2^).

#### DON reduces duodenal villus height

Results as presented in Table 2 show a significant shortening of the villi in the duodenum for the DON group compared to the control group (*P* = 0.014). A trend was observed for reduction in the villus height to crypt depth ratio in the duodenum (*P* = 0.073) and for the crypt depth in the jejunum in the DON group (*P* = 0.052).

**Table 2:** Effect of DON on villus height and crypt depth measurements.

	control diet	DON diet	*P*
**mid-duodenum**			
villus height (µm)	2,175±26.8	2,010±52.9	0.014 (*)
crypt depth (µm)	143±4.7	154±9.1	0.269
villus to crypt ratio	16±0.5	13±1.0	0.073
**mid-jejunum**			
villus height (µm)	894±72.6	792±61.6	0.303
crypt depth (µm)	178±12.3	150±6.4	0.052
villus to crypt ratio	5±0.3	5±0.4	0.978
**mid-ileum**			
villus height (µm)	711±63.8	689±23.6	0.748
crypt depth (µm)	153±10.6	144±6.7	0.456
villus to crypt ratio	5±0.2	5±0.3	0.487

Analysis was based on 9 animals per treatment, and the mean of 5 to 15 measurements per segment per animal was calculated; data are presented as weighted mean ± SEM.

(*) significantly different (P<0.05).

### *In vitro* experiment

#### No impact of DON on *C. perfringens* growth, alpha toxin production and *netB* transcription

**Figure 3:**
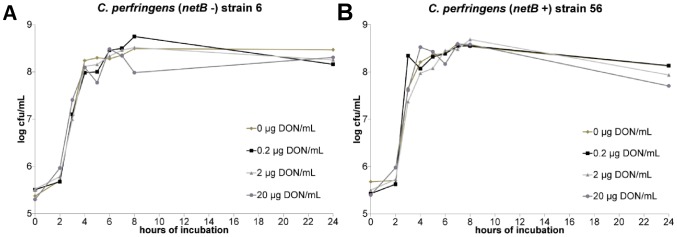
No impact of DON on *in vitro* growth of *C. perfringens*. *C. perfringens* strains 6 (a) and 56 (b) were grown in TGY broth medium containing 0, 0.2, 2 or 20 µg DON/mL. Samples were taken at 0, 2, 3, 4, 5, 6, 7, 8 and 24 h after inoculation with an overnight culture of *C. perfringens.* The number of colony forming units (cfu) per mL was determined by bacterial plating of 10-fold dilutions. Results are presented as the mean cfu/mL. There is no significant difference between the different test conditions.

**Figure 4:**
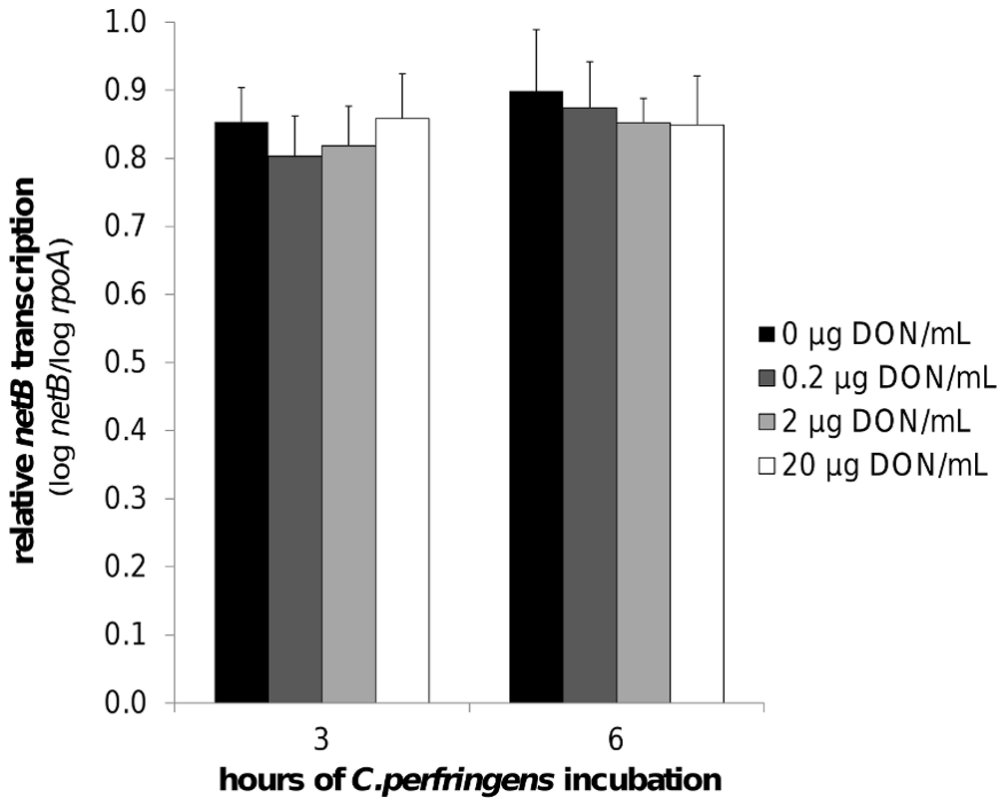
*NetB* toxin transcription is not influenced by DON. Transcription level of *netB* toxin was analysed by qRT-PCR of *C. perfringens* strain 56 RNA samples collected from *in vitro* culture material in the mid (after 3 h incubation) and late logarithmic (after 6 h incubation) growth phase. *C. perfringens* strain 56 was grown in absence or presence (0.2, 2, 20 µg/mL) of DON. The results for the *netB* gene transcription were normalized to the *rpoA* gene transcription. Results are presented as the mean value of three biological replicates. Error bars represent SD. There is no significant difference between the different test conditions.

The results of the *C. perfringens* growth assay showed no influence of 0, 0.2, 2 or 20 µg DON/mL on the bacterial growth curve (Figure 3 a, b). Quantification of alpha toxin also revealed no impact of these concentrations of mycotoxin. The mean OD of the alpha toxin detection, relative to the positive control value was 1.1±0.05, 1.1±0.01, 1.1±0.03 and 1.1±0.03 in the presence of 0, 0.2, 2 or 20 µg DON/mL, respectively. Measurement of the *C. perfringens* transcription level of *netB* by qRT-PCR showed no influence of DON (Figure 4).

## Discussion

Our data demonstrate that the mycotoxin DON is a predisposing factor for the development of NE in broiler chickens. Indeed, contamination of the diet with DON at concentrations below the EU maximum guidance level of 5,000 µg/kg feed, significantly increased the number of chickens affected with NE.

The distribution of NE lesions in the present infection study, mainly in duodenum and jejunum, is similar as in a previously described NE infection trial, where coccidiosis was included as predisposing factor [32]. The proximal part of the intestinal tract is the main absorption site for DON [22], [28], [38]. Proximal intestinal epithelial cells are thus exposed to high concentrations of DON following ingestion of DON-contaminated feed, and are as such sensitive due to their high protein turnover [22], [39], [40]. DON negatively affected the proximal part of the intestinal tract, demonstrated by the significantly reduced villus height in the duodenum. These results are in accordance with those observed by Awad *et al.* [28], who tested a similar contamination level and duration of exposure of DON. The decreased villus height will compromise the effectiveness of nutrient absorption due to the decreased absorption surface area [27]. Enterocytes must differentiate during their migration along the crypt-villus axis to fully express their digestive functions [41]. The sucrase and maltase activities increase for example towards the villus tip in chicks [42]. As such, the negative impact of DON on the villus height can be associated with an impaired nutrient digestion due to a reduced number of differentiated epithelial cells [27].

DON also modulates the intestinal paracellular transport leading to an increased passage of macromolecules and bacteria [26]. The intestinal barrier function is maintained by intercellular structures, including tight junctions, adherence junctions and desmosomes [25], [40]. The TEER is considered as an indicator of the epithelial integrity and thus of the organization of tight junctions. In accordance with literature [26], [39], we demonstrated a reduction of the TEER of the duodenal epithelium after DON exposure. These toxic effects on epithelial cells contribute to an increased protein availability in the intestinal lumen due to leakage of plasma amino acids or proteins into the gut. Consequently, this creates an environment that favors for massive overgrowth of *C. perfringens*. Indeed, in this study, the total duodenal protein level was increased. This could be caused by malabsorption, a negative effect on nutrient digestion or plasma amino acid or protein leakage in the intestine due to the altered intestinal barrier integrity. Malabsorption and maldigestion was also suggested by the decreased duodenal villus height. Furthermore, it has been shown that DON selectively modulates the activities of different intestinal transporter proteins for nutrients, and negatively influences the sodium associated amino acid co-transport for serine and proline, leading to an increased intestinal content of these amino acids [39], [43], [44]. We propose a negative effect of DON on the small intestinal mucosa that leads to malabsorption, maldigestion and leakage of plasma amino acids or proteins into the intestinal lumen, which provide the necessary growth substrate for extensive proliferation of *C. perfringens*.

The *in vitro* growth of *C. perfringens* was not affected by concentrations of DON up to 20 µg/mL. No influence on alpha toxin production, and *netB* transcription was demonstrated. These results suggest that the observed predisposing effect is due to the toxic effect of DON on the animal host rather than its effect on the bacterium itself.

**Figure 5:**
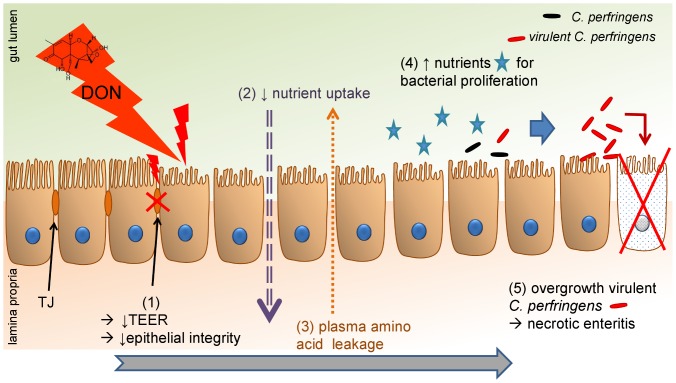
Deoxynivalenol predisposes for *C. perfringens* induced necrotic enteritis. DON decreased villus height and reduced transepithelial electrical resistance (1), leading to a decreased absorption and digestion of dietary nutrients; and an increased intestinal barrier permeability, respectively. Taken together with an increased intestinal protein level, these results suggest an impaired nutrient uptake (2) and leakage of plasma amino acids (3) into the intestinal lumen, providing the necessary growth substrate for *C. pefringens* proliferation (4). Proliferation of virulent (*netB* positive) *C. perfringens* induces necrotic enteritis (5).

In conclusion, as summarized in Figure 5, our results indicate that the intake of DON contaminated feed at contamination levels below the EU maximum guidance level, is a predisposing factor for the development of necrotic enteritis in broiler chickens due to the negative influence on the epithelial barrier, and to an increased intestinal nutrient availability for clostridial proliferation. We showed that DON has a cytotoxic effect on enterocytes, leading to an altered intestinal barrier function, resulting in an increased permeability of the intestinal wall. Additionally, the shortened villus height could lead to a decreased absorption of dietary proteins, resulting in an increased protein concentration in the intestinal lumen. These mechanisms lead to an increased protein content in the intestinal lumen, which is available for clostridial proliferation resulting in the development of necrotic enteritis.

## Supporting Information

Table S1 **Different mycotoxins tested, their limit of detection (LOD) and limit of quantification (LOQ) and their concentration in the different feeds.**
(XLSX)

Table S2 **Primer sequences used for qRT-PCR transcription analysis of** ***netB*** **toxin. Sequences are presented from 5′ to 3′.**
(PDF)
