# Supplementary material for: The Mycotoxin Deoxynivalenol Predisposes for the Development of Clostridium perfringens-Induced Necrotic Enteritis in Broiler Chickens
Source: PLoS One. 2014 Sep 30;9(9):e108775. doi: 10.1371/journal.pone.0108775 (PMC4182565; doi:10.1371/journal.pone.0108775)
Supplement: Table S2 — Primer sequences used for qRT-PCR transcription analysis of netB toxin. Sequences are presented from 5′ to 3′. (PDF) [file pone.0108775.s002.pdf]

Table S2: Primer sequences used for qRT-PCR transcription analysis of *netB* toxin. Sequences are presented from 5' to 3'.

target	forward primer	reverse primer	analysis	size (nt) of amplified products
netB	TGA TAC CGC TTC ACA TAA AGG T	ACC GTC CTT AGT CTC AAC AAA T	standard PCR	448
netB	TCA ATT GGT TAT TCT ATA GGC GGT A	ATA TGA AGC ATT TAT TCC AGC ACC A	qPCR	75
rpoA	ACA TCA TTA GCG TTG TCA GTT AAA G	GAG GTT ATG GAA TAA CTC TTG GTA ATG	standard PCR	613
rpoA	CCA TCT GTT TTT ATA TCT GCT CCA GTA	GGA AGG TGA AGG ACC AAA AAC TAT T	qPCR	81
